# Application of gold immunochromatographic assay strip combined with digital evaluation for early detection of *Toxoplasma gondii* infection in multiple species

**DOI:** 10.1186/s13071-024-06180-1

**Published:** 2024-02-22

**Authors:** Jiyuan Fan, Hao Sun, Jiawen Fang, Yafan Gao, Haojie Ding, Bin Zheng, Qingming Kong, Xunhui Zhuo, Shaohong Lu

**Affiliations:** 1 School of Basic Medical Sciences and Forensic Medicine, Hangzhou Medical College, Hangzhou, 310013 China; 2 Engineering Research Center of Novel Vaccine of Zhejiang Province, Hangzhou Medical College, Hangzhou, China; 3 Key Laboratory of Bio-Tech Vaccine of Zhejiang Province, Hangzhou Medical College, Hangzhou, China

**Keywords:** *T. gondii*, GICA, AMA1C, Early detection, Digital application

## Abstract

**Background:**

Timely diagnosis of *Toxoplasma gondii* infection is necessary to prevent and control toxoplasmosis transmission. The gold immunochromatographic assay (GICA) is a means of rapidly detecting pathogen in samples. GICA-based diagnostic methods have been developed to accurately detect pathogens with high sensitivity and specificity, and their application in *T. gondii* diagnosis is expected to yield good results.

**Methods:**

Colloidal gold test strips were produced using *T. gondii* C-terminal truncated apical membrane antigen 1 (AMA1C). Colloidal gold-AMA1C and colloidal gold-murine protein conjugate were synthesized under optimal conditions. A nitrocellulose membrane was treated with AMA1C and goat anti-mouse antibody as the test line and control line, respectively. In total, 90 cat serum samples were tested using AMA1C-GICA and a commercial enzyme linked immunosorbent assay (ELISA) kit. The GICA results were digitally displayed using a portable colloidal gold immunochromatographic test strip analyzer (HMREADER). The sensitivity, specificity, and stability of AMA1C-GICA were assessed, and this was then used to examine clinical samples, including 203 human sera, 266 cat sera, and 81 dog sera.

**Results:**

AMA1C-GICA had a detection threshold of 1:32 for *T. gondii*-positive serum. The GICA strips specifically detected *T. gondii* antibodies and exhibited no reactivity with *Plasmodium vivax*, *Paragonimus kellicotti*, *Schistosoma japonicum*, *Clonorchis sinensis*, and *Schistosoma mansoni*. Consequently, 15 (16.7%) positive samples were detected using the AMA1C-GICA and commercial ELISA kits for each of the assays. The receiver-operating characteristic curve showed that GICA had a relative sensitivity of 85.3% and specificity of 92%, with an area under the curve of 98%. After analyzing clinical samples using HMREADER, 1.2%–23.4% of these samples were found to be positive for *T. gondii*.

**Conclusions:**

This study presents a novel assay that enables timely and efficient detection of serum antibodies against *T. gondii*, thereby allowing for its early clinical diagnosis. Furthermore, the integration of digital detection using HMREADER can enhance the implementation of GICA.

**Graphical Abstract:**

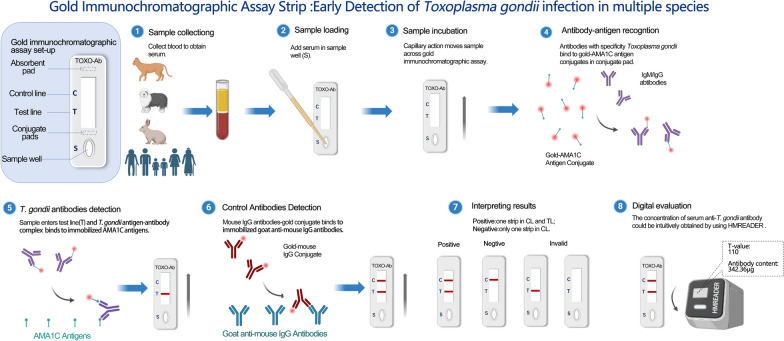

**Supplementary Information:**

The online version contains supplementary material available at 10.1186/s13071-024-06180-1.

## Background

*Toxoplasma gondii*, an intracellular protozoan apicomplexan, was initially observed by researchers in North Africa approximately 115 years ago [1]. *Toxoplasma gondii* has a wide host range, infecting > 200 species of warm-blooded creatures, including humans [2]. *Toxoplasma gondii* may cause encephalitis, hydrocephalus, and retinal choroiditis [3], which can be fatal for people with compromised immune systems [4]. The global seropositivity rate for *T. gondii* has been estimated to be 25.7% [5, 6]. In addition, the seroprevalence of *T. gondii* in cats and dogs varies from 4 to 40% [7–9] and from 10 to 70% [10–12], respectively. Thus, the persistence of *T. gondii* infection poses a significant public health concern worldwide. Therefore, a rapid and accurate diagnostic test is required for *T. gondii* in clinical settings.

Currently, pathological, immunological, and molecular techniques are used for detecting *T. gondii* [13]. Primary hospitals are recommended not to use pathogenicity testing, especially direct microscopy, as these methods are time consuming and have low positive detection rates [14]. In addition to the conventional parasitological pathogenetic testing, multiple alternative tests are available for determining *T. gondii* infection, with enzyme-linked immunosorbent assay (ELISA) being the most widely employed method [15]. Serological testing is the preferred approach in most epidemiological studies focused on toxoplasmosis [16]. However, differentiating between cases with weak-positive ELISA results may be challenging, thereby leading to the emergence of “gray zone” scenarios that necessitate supplementary validation in combination with western blotting [15, 17]. The gold immunochromatographic assay (GICA) strips utilize distinctive antigen–antibody immunoreactivity to facilitate rapid detection of *T. gondii* in various samples. Compared with existing laboratory-based diagnostic platforms, GICA provides a range of testing scenarios and result visualization options, enabling prompt testing by individuals without specialized training [18]. Colloidal gold immunochromatography offers several advantages compared with ELISA, including faster detection times, simpler procedures, and greater sensitivity.

*Toxoplasma gondii* apical membrane antigen 1 (AMA1) is a microneme protein that plays a crucial role in both detection of *T. gondii* infection and parasite adhesion to host cells for subsequent entry to promote tachyzoite replication [6, 18, 19]. AMA1 enables *T. gondii* to infiltrate host cells despite its incomplete functionality [20]. The use of AMA1 in feline specimens favorably coincides with the commercially available *T. gondii* IgG antibody test kit, and ELISA technique, employing recombinant AMA1 proteins, which exhibit notable sensitivity and specificity for *T. gondii* detection in murine as well as human sera [20, 21]. These findings indicate that AMA1 is a promising candidate for the development of diagnostic assays aimed at early identification of *T. gondii* invasion [20].

In this study, ELISA and GICA strips were generated using C-terminal truncated AMA1 (AMA1C) and compared. Serum samples from several species were examined using the standard procedure. Using inexpensive digitization tools that help in obtaining semiquantitative results, we demonstrated the ease, speed, and specificity of our method for point-of-care testing (POCT).

## Methods

### Animals and samples

New Zealand rabbits were purchased from Zhejiang Provincial Laboratory Animal Center. Sera positive for *Plasmodium vivax*, *Paragonimus kellicotti*, *Schistosoma japonicum*, *Clonorchis sinensis*, and *Schistosoma mansoni* [22, 23] were maintained in our laboratory. In total, 203 human serum samples were donated by the Second People’s Hospital of Lishui. Moreover, 347 clinical serum samples were collected from healthy cats and dogs in Jiaxing and Wenzhou, Zhejiang Province, China. All domestic cats and dogs were provided with good food and comfortable living conditions by their owners. After rescue, the stray cats were provided with ample food and a comfortable living environment. Serum samples were stored at − 80 °C.

### *T. gondii* RH strains and cell culture

High-virulence *T. gondii* RH strains (genotype I; ATCC, USA) were maintained in human foreskin fibroblasts (HFFs; ATCC, USA) in complete Dulbecco’s modified Eagle medium (DMEM; Gibco™, China) supplemented with 10% fetal bovine serum (FBS; Gibco™, USA) and 1% penicillin-streptomycin solution (100×; Thermo Fisher Scientific, USA) at 37 °C in a 5% CO_2_ incubator.

### Production of anti-*T. gondii* antibody in rabbit serum samples

Four rabbits (two in each group) were intraperitoneally injected with sterile phosphate buffer saline (PBS; Biosharp, China) and 1 × 10^6^/100 µl RH strain tachyzoites. Serum samples were collected every other day from 1 to 31 days post infection (dpi) and kept at − 80 °C until use.

### Anti-serum against *T. gondii* was purified via caprylic acid/ammonium sulfate precipitation (CAAS)

Rabbit anti-serum against *T. gondii* was purified via the CAAS method as previously described [24, 25]. Each aliquot of anti-serum (5 ml) was diluted threefold with acetate buffer (pH: 4.0) and adjusted to a pH value of 4.6 using sodium hydroxide (0.1 mol/l). Octanoic acid (Sigma-Aldrich, Germany) (170 μl) was added dropwise under continuous stirring. Centrifugation was performed at 10,000*g* for 30 min to separate the precipitate containing non-IgG proteins. The supernatant was dialyzed in 500 ml 0.01 M PBS overnight at 4 °C to pH 7.5. The IgG was then precipitated in 0.01 M PBS by adding saturated ammonium sulfate (20 ml) with stirring. The obtained precipitate was dialyzed with 0.01 M PBS (500 ml) overnight at 4 °C after being dissolved in PBS to its original volume (5 ml).

### Construction of expression plasmids

The pET-32a (+)-AMA1 and pET-30a (+) (Novagen, Germany) expression plasmids were kept in our laboratory. Amino acids 67–287 of AMA1 were selected for the production of AMA1N, and amino acids 287–569 of AMA1 were selected for the production of AMA1C [20]. Specific primers (Additional file 1: Table S1) were designed to amplify the gene products via PCR. The EcoRV and SalI digestion sites on the pET-30a (+) plasmid were used to clone the PCR products, and pET-30a-AMA1, pET-30a-AMA1N, and pET-30a-AMA1C were generated.

### Antigen expression, purification, and renaturation

pET30a-AMA1C, pET30a-AMA1N, and pET30a-AMA1 were separately transformed into the competent *Escherichia coli* strain Rosetta (DE3) pLysS (Novagen, Germany). They were then cultured overnight on lysogeny broth (LB) agar plates. A single colony was subcultured in LB medium, and protein expression was stimulated for 18 h at 23 °C by adding 1.0 mM isopropyl-d-1-thiogalactopyranoside (Aladdin, China). AMA1, AMA1N, and AMA1C were purified as previously described [20]. The three proteins were complexed for 24–36 h at 4 °C with renaturation buffers (pH 8.0–9.0; 0.1 M Tris–HCl, 0.5 mM L-Arg, 1 mM oxidized glutathione, and 1 mM reduced glutathione). Expression and purity of AMA1, AMA1N, and AMA1C were analyzed using sodium dodecyl sulfate-poly acrylamide gel electrophoresis (SDS-PAGE). The immunogenicity of proteins was subsequently assessed via western blotting by incubating the proteins with mouse His-tag monoclonal antibody (Proteintech Group, USA) (1:5000). Membranes were developed using BeyoECL Star (Beyotime Biotechnology, China) and visualized for acquisition using the ChemiDoc™ chemiluminescence system (Bio-Rad, Hercules, CA, USA). The concentrations of the purified proteins were quantified using a Bradford Protein Assay Kit (P0006, Beyotime Biotechnology, China).

### Determination of the optimal labeling protein

AMA1, AMA1N, and AMA1C were incubated with goat anti-rabbit IgG H&L (HRP) (Abcam, ab205719) for 1 h. Membranes were exposed using BeyoECL Star.

A 96-well flat-bottom microtiter plate (BKMAM, China) was coated with AMA1, AMA1N, and AMA1C (100 μl each) and incubated overnight at 4 °C. The unbound protein was discarded, and the plate was washed three times with Tween-20 in PBS (PBST). The plate was blocked with 2% bovine serum albumin (BSA; Sigma-Aldrich, Germany) for 1 h and then washed three times with PBST before incubating it with rabbit serum diluted with PBST at a 1:200 ratio for 1 h at 37 °C. After washing, the wells were incubated with goat anti-rabbit IgG H&L (HRP) (Abcam, ab205719) and IgM mu chain (HRP) (Abcam, ab97195) diluted with PBST at 1:10,000 for 2 h at 37 °C. Following a final wash, the plate was incubated with 100 μl TMB (Beyotime Biotechnology, China) for 15 min at 37 °C. After stopping the test with 50 μl of 2 M H_2_SO_4_, the absorbance at 450 nm was measured with a Bio-Tek synergy 2 microplate reader (Bio-Tek, USA); each parallel well in the two serum samples was read twice.

### Preparation of colloidal gold mouse IgG and AMA1C conjugate

Colloidal gold solution (1 ml) (NG20-B100, Bioassay works, USA) was added to Eppendorf (EP) tubes and adjusted to the optimal pH with 0.2 M K_2_CO_3_ (0, 2, 4, 6, 8, 10, 12, 14, 16, and 18 μl) and mixed with 100 μl of mouse IgG (Beyotime Biotechnology, China) or AMA1C that had been diluted with PBS (0.01 M, pH 7.4) at 100 μg/ml with slight shaking for 5 min. Each 1 ml of colloidal gold solution was then mixed with 100 μl of a 10% NaCl solution for another 10 min. The color of the mixture changed from blue to bright red after 1 h incubation at room temperature as the pH increased, and the optimum pH of proteins for colloidal gold conjugation was a minimum pH of red invariant.

Colloidal gold solution (1 ml) was adjusted to the optimum pH, and 100 μl of mouse IgG (0, 2, 4, 6, 8, 10, 12, 14, 16, 18, and 20 μg/ml) or AMA1C (0, 1, 2, 3, 4, 5, 6, 7, 8, 9, and 10 μg/ml) was added with slight shaking for 10 min. Then, NaCl solution (100 μl, 10%) was added to each 1 ml of colloidal gold solution for further 10 min. The absorption at 525 nm was measured.

Colloidal gold solution (10 ml) was added and adjusted to the optimal pH value with 0.2 M K_2_CO_3_. Then, the optimal mouse IgG or AMA1C labeled concentration was added. After 10 min of vortex mixing, 1 ml 10% BSA (Sigma-Aldrich, Germany) solution was added to block the unreacted sites of the gold nanoparticles and then placed in the vortex mixer for 1 h. After removing the supernatant, the precipitate was redissolved in 1 ml dilution buffer [0.002 M Tris buffer (pH 8.0)] containing 20% sucrose (Beyotime Biotechnology, China), 5% trehalose (Sigma-Aldrich, Germany), and 1% BSA solution and stored at 4 ℃.

### Preparation of AMA1C-GICA strips

**Fig. 1:**
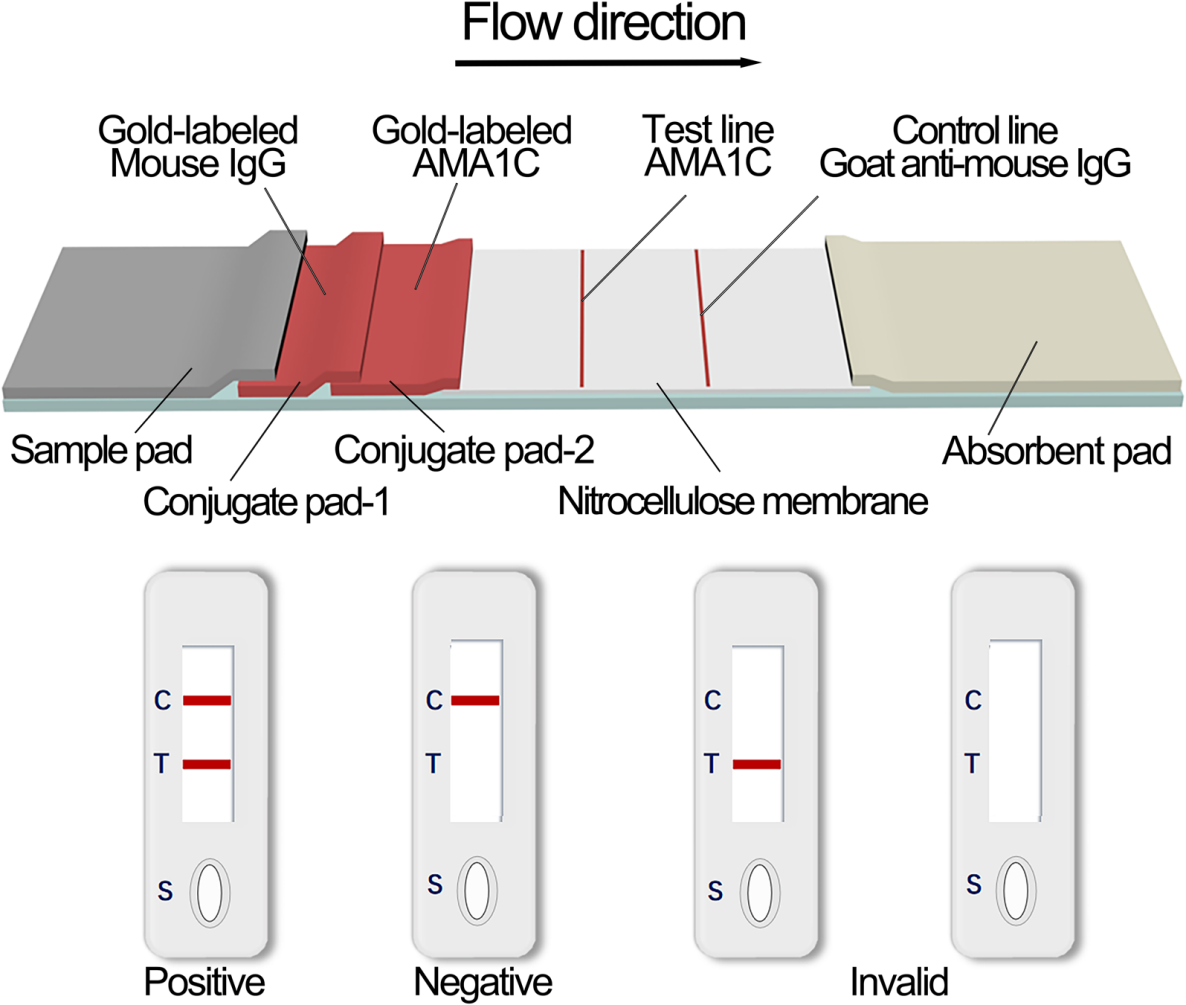
Schematic representation of the design of gold immunochromatographic assay strip, negative, positive, and invalid results. CL represents the control line; TL represents the test line

A glass fiber (Kinbio, RB65, China) was used as the sample pad. AMA1C and goat anti-mouse IgG (H&L) (Solarbio, China) were diluted to a concentration of 1 mg/ml in 0.01 M PBS with 3.0% trehalose. Using the Biodot XYZ3050TM Dispense System (BioDot, XYZ3050TM, United States), the diluted proteins (1 mg/ml) were deposited onto a nitrocellulose (NC) membrane (Sartorius, CN140, Germany) at 1 μl/cm to form the test line (TL) and control line (CL), respectively. Colloidal gold probe was sprayed onto the polyester fiber (Kinbio, DL98, China), which was used as the conjugate pad. The GICA strip, which includes the sample pad, conjugate pad, NC membrane, and absorbent pad, was described and assembled as shown in Fig. 1. A programmable slitter (AUTOKUN, HGS201, China) was used for constructed PVC plate, which was divided into 4-mm-wide test strips and placed in a sealed bag for later use.

### Sensitivity, specificity, and stability of the AMA1C-GICA strips

The visual limit of the AMA1C-GICA strips was evaluated using *T. gondii*-positive serum diluted to various concentrations (undiluted, 1:2, 1:4, 1:8, 1:16, 1:32, 1:64, 1:128, and 1:256). The specificity of the strips was used to test the positive sera of *P. vivax*, *P. kellicotti*, *S. japonicum*, *C. sinensis*, *S. mansoni*, and *T. gondii*. The results were observed after dropping the sera on the detection end of the test strips from the same batch of test strips to assess the specificity of the method. The negative and blank controls were *T. gondii*-negative serum (100 µl) and 0.01 M PBS (pH 7.4), respectively. The AMA1C-GICA strips were stored at 4 °C (12 weeks) and 37 °C (4 weeks) to evaluate the stability with *T. gondii*-positive serum sample. *Toxoplasma gondii*-positive serum samples were deposited on the detecting end of the same batch of the produced test strips, and the outcomes were examined to assess the stability of the method. *Toxoplasma gondii*-negative serum samples were used as negative controls.

### Inter- and intra-batch variation of the GICA strips

A minimum of two or more groups of test strips from different batches were selected, and then three from each group were randomly selected to determine the inter- and intra-batch variation of colloidal gold test strips using rabbit serum that was positive/negative for *T. gondii*.

### Standard curve for colloidal gold digitization

Purified antibodies were diluted at various concentrations (240, 180, 120, 60, 40, 12, 1.2 μg/100 μl) to establish the standard curve with a portable colloidal GICA strip analyzer (HMREADER). HMREADER was found to be capable of analyzing the grayscale values of the C/T lines present on strips using its internal optical components. By using a standard curve and calculation formula, it was able to automatically perform data calculations (e.g. for T-values) and relative quantification. Strips from the same lot were used, and the detection end of the specimen received sequential additions of 100 μl of diluted purified antibody and *T. gondii*-negative serum. After 15 min, the strips were read with HMREADER. A linear regression curve was fitted using HMREADER (software version: v8.5.1715) based on the known mass of the purified antibody and the measured T-value.

### Conformity testing and clinical serum sample testing

In total, 90 cat serum samples were assessed using the AMA1C-GICA strips and a commercial ELISA kit (HT8012, Haitai, China) to evaluate the accuracy of the strips [2]. A total of 60 rabbit serum samples were tested with the AMA1C-GICA strips and commercial ELISA kits. Colloidal gold results were digitally analyzed using HMREADER. All clinical serum samples (including animals and humans) were analyzed using the AMA1C-GICA strips, followed by antibody concentration determination using the HMREADER within 15 min.

### Statistical analysis

Graphs were plotted using GraphPad Prism version 9.5. Statistical analysis and calculation of the area under the curve (AUC) of the receiver-operating characteristic (ROC) curve were performed using SPSS software version 27.0. A *P*-value < 0.05 was considered statistically significant. HMREADER (v8.5.1715) was used for the digital reading of the test strips.

## Results

### Production of AMA1, AMA1N, and AMA1C antigens

**Fig. 2:**
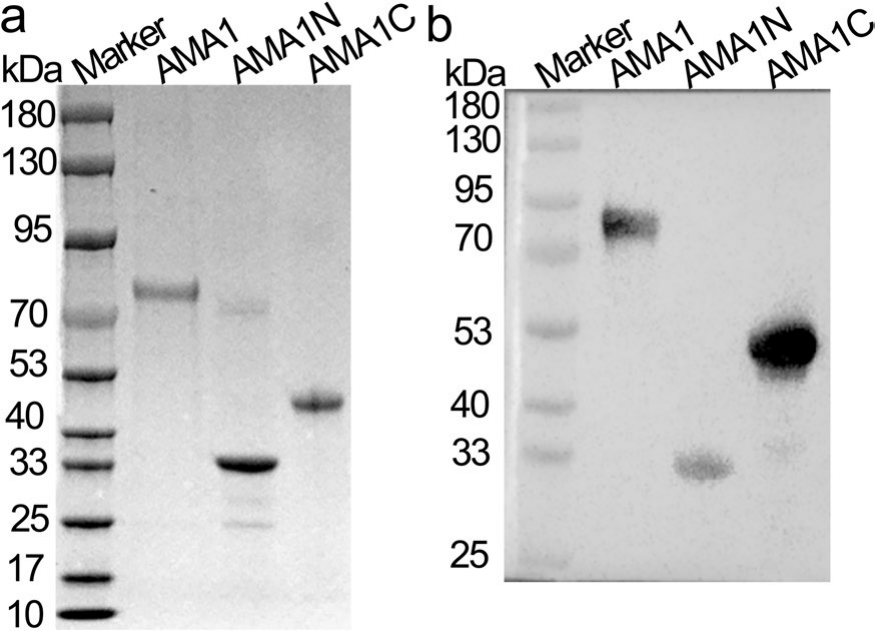
Expression and analysis of variants of AMA1 protein (AMA1N, AMA1C, and full-length AMA1). **a** Sodium dodecyl sulfate-poly acrylamide gel electrophoresis (SDS-PAGE) analysis of full-length AMA1, AMA1N, and AMA1C. **b** Western blot analysis of full-length AMA1, AMA1N, and AMA1C with His-tag

AMA1 protein variants (AMA1N, AMA1C, and full-length AMA1) were produced as insoluble proteins and were purified with a Ni‐NTA Sefinose™ Resin column (Sangon Biotech, China). The purity of the three proteins was assessed with SDS-PAGE after denaturation (Fig. 2a). The immunoreactivity of AMA1 protein variants was confirmed via western blotting (Fig. 2b).

### Determination of positive and negative sera for anti-*T. gondii* antibodies

**Fig. 3:**
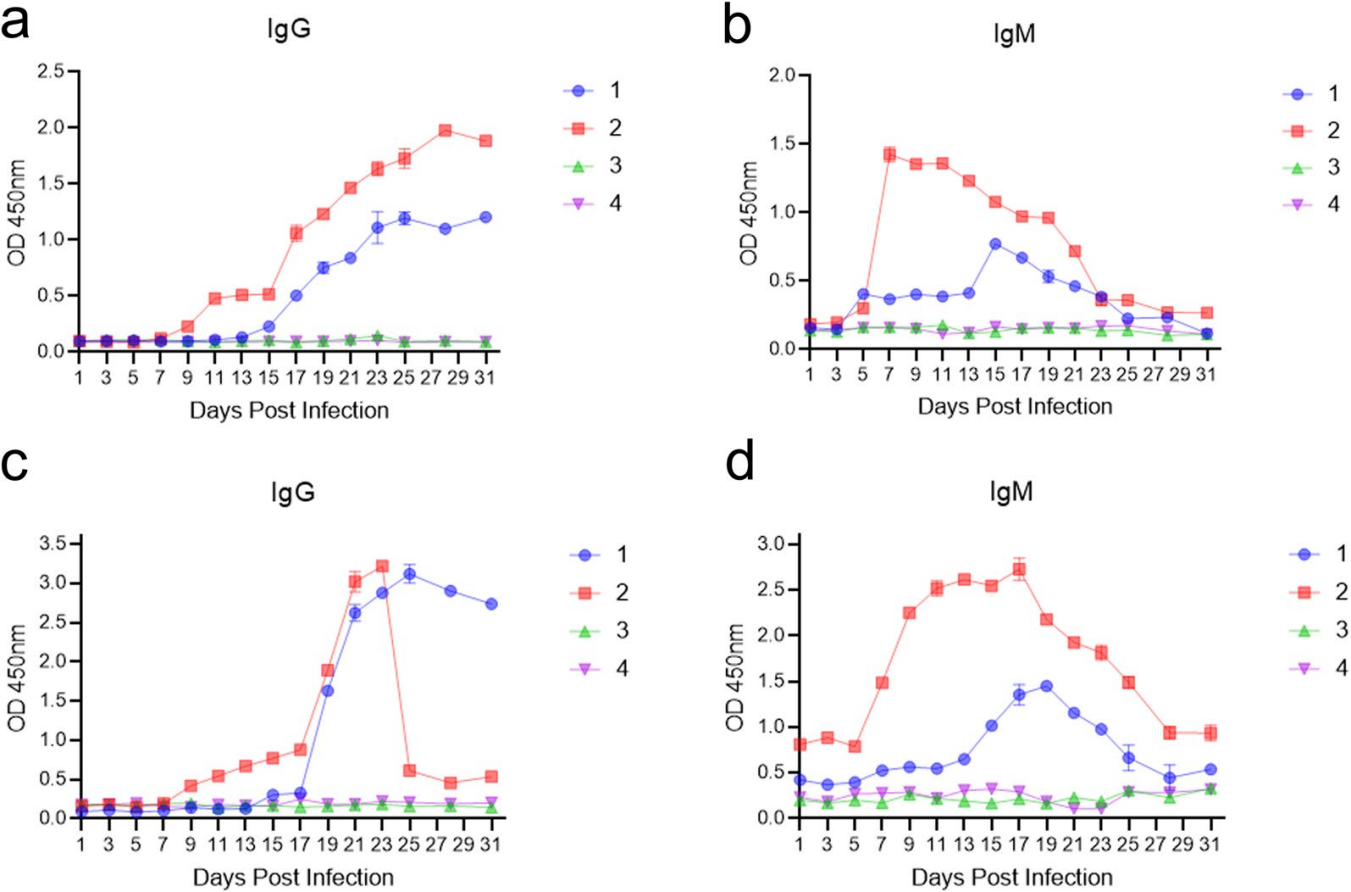
ELISA of *Toxoplasma gondii* antibodies in the sera of rabbits from 1 to 31 dpi. **a**, **b** The *T. gondii* Antibody (IgG and IgM) Diagnostic Kit was used to determine negative and positive anti-*T. gondii* antibodies in the sera of four rabbits. **c**, **d** AMA1C-ELISA of *T. gondii* antibodies (IgG and IgM) in the sera of rabbits from 1 to 31 dpi. 1–4: represent four rabbits, where 1 and 2 were injected with 10^6^ RH strain and 3 and 4 with negative controls

A *T. gondii* antibody (IgG) diagnostic kit and a *T. gondii* antibody (IgM) diagnostic kit (GIVEI, China) were used to determine negative and positive anti-*T. gondii* antibodies in the sera of four rabbits [26]. The first rabbit was found to be seropositive for anti-*T. gondii* antibodies (IgG) at 17 dpi and the second rabbit at 11 dpi (Fig. 3a). In addition, the first rabbit was found to be seropositive for anti-*T. gondii* antibodies (IgM) at 15 dpi and the second rabbit at 7 dpi (Fig. 3b). The *T. gondii* antibody diagnostic kits both detected 18 *T. gondii*-positive serum samples.

### AMA1C was identified as the optimal candidate for colloidal gold strips

*Toxoplasma gondii* sera were used for screening the optimal marker protein. The negative sera of both AMA1-ELISA and AMA1N-ELISA showed false-positive results (Additional file 1: Table S2). AMA1C was specifically recognized by polyclonal antibodies in rabbit serum, suggesting that AMA1C is sufficiently immunogenic compared with full-length AMA1 and AMA1N proteins. Furthermore, AMA1C reacted not only with IgG but also with IgM (Fig. 3c and d) and was selected as the optimal candidate for colloidal gold strips.

### Optimal pH and concentration of conjugate proteins

**Fig. 4:**
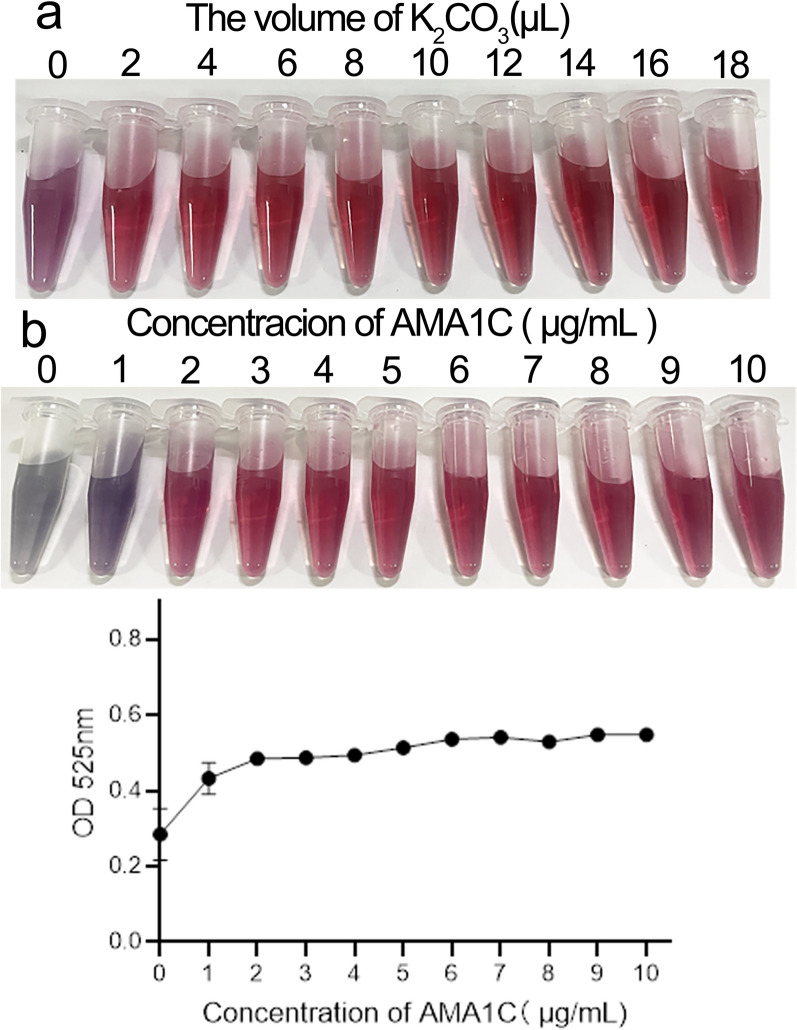
The optimum pH and conjugate concentrations of AMA1C. **a** The colloidal gold solution was adjusted to different pH values using 0.2 M K_2_CO_3_ to obtain optimum pH value of AMA1C. **b** An increasing amount of AMA1C was added to colloidal gold solution to obtain optimal conjugate amount of AMA1C by direct observation or by using a Multifunctional Enzyme Labeler

According to the isoelectric point of mouse IgG, a pH of approximately 8 (8 μl 0.2 M K_2_CO_3_) was appropriate for conjugation with the colloidal gold solution of red invariant, and the minimum concentration of colloidal gold-labeled mouse protein of maximum absorbance value was 2 μg/ml (Additional file 2: Fig. S1). Therefore, the optimum pH was approximately 8, and the optimum concentration was 2 μg/ml according to the detailed description in the method. According to the isoelectric point of AMA1C, a pH of approximately 8 was appropriate for conjugation with the colloidal gold solution of red invariant, and the minimum murine protein concentration labeled with colloidal gold of maximum absorbent value was 6 μg/ml (Fig. 4).

### Sensitivity, specificity, and stability of the AMA1C-GICA strips

**Fig. 5:**
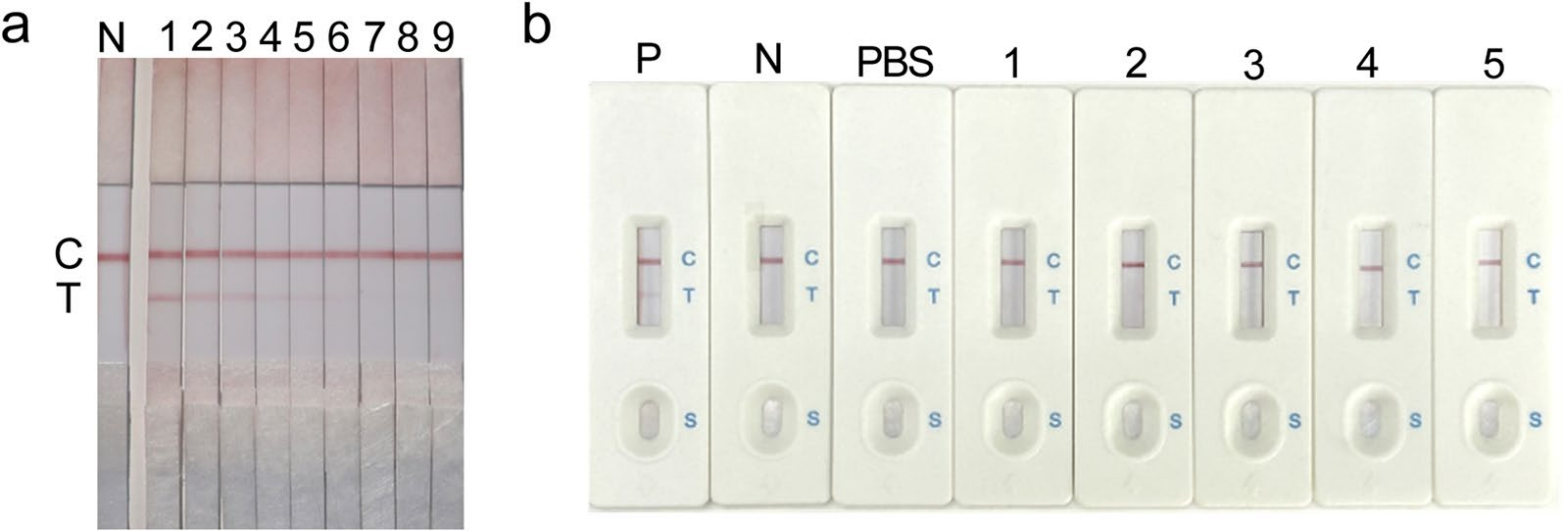
The sensitivity and specificity of AMA1C-GICA strips. **a** Sensitivity of gold immunochromatographic assay (GICA) strip based on AMA1C. N: *T. gondii*-negative control; 1–9: *T. gondii*-positive serum ratio dilution (undiluted, 1:2, 1:4, 1:8, 1:16, 1:32, 1:64, 1:128, and 1:256). **b** Specificity of GICA strip based on AMA1C. P: *T. gondii*-positive control; N: *T. gondii*-negative control; 1–5: Positive serum for *Plasmodium vivax*, *Paragonimus kellicotti*, *Schistosoma japonicum*, *Clonorchis sinensis*, and *Schistosoma mansoni*

Serial dilutions of *T. gondii*-positive rabbit standard sera were prepared at 1:256 dilution, and susceptibility testing was performed using colloidal gold test strips from the same lot. When diluted to 1:32, a slight banding of the T-line could still be observed. Therefore, the visual limit of detection (vLOD) of the GICA strips was 1:32 (Fig. 5a).

Positive standard sera for *T. gondii*, *P. vivax*, *P. kellicotti*, *S. japonicum*, *C. sinensis*, and *S. mansoni* were tested with colloidal gold test strips from the same lot. The strips for detection of *T. gondii*-positive serum samples presented both TL and CL, whereas the strips tested with other positive sera only presented CL (Fig. 5b). The gold strips were therefore specific for anti-*T. gondii* sera among the other sera tested.

The GICA strips were stored at 4 °C for 12 weeks and at 37 °C for 4 weeks and examined with *T. gondii*-positive and negative rabbit serum samples to evaluate their stability. The AMA1-GICA strips stored under both conditions still detected the *T. gondii*-positive serum samples that presented the CL and TL (Additional file 2: Fig. S2).

### Inter- and intra-batch variation of the AMA1C-GICA strips

Three different batches of test strips were selected, with three strips randomly selected from each batch. Each of the nine test strips was tested with a 1:10 dilution of *T. gondii* serum. The *T. gondii*-positive serum sample presented both the CL and TL, whereas negative serum only presented the CL (Additional file 2: Fig. S3).

### Standard curve determination for colloidal gold digitization

**Fig. 6:**
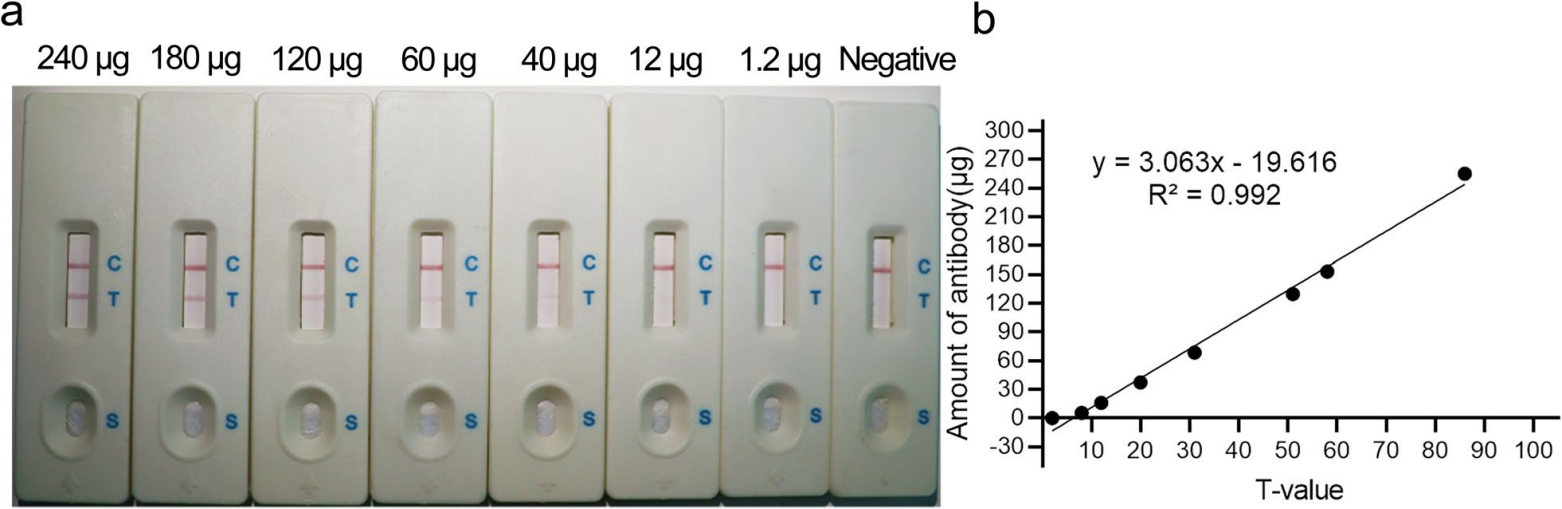
The standard curve for colloidal gold digitization. **a** A schematic diagram of colloidal gold test strips with different concentrations of purified antibodies. **b** The standard curve for colloidal gold digitization

Depending on the amount of the serum sample, a change in the gradient of the TL color of the test strip was clearly observed (Fig. 6a, b). The limit of detection of HMREADER was determined. A linear regression equation (*R*^2^ = 0.992) was used to calculate the *T*-value of 12, which corresponds to an antibody level of 15.78 µg. A positive result was defined as a *T*-value of > 12 and a negative result as a *T*-value of < 12. A *T*-value of 12 was considered suspicious, and the test should be repeated.

### Clinical test of the newly developed colloidal gold strip

**Fig. 7:**
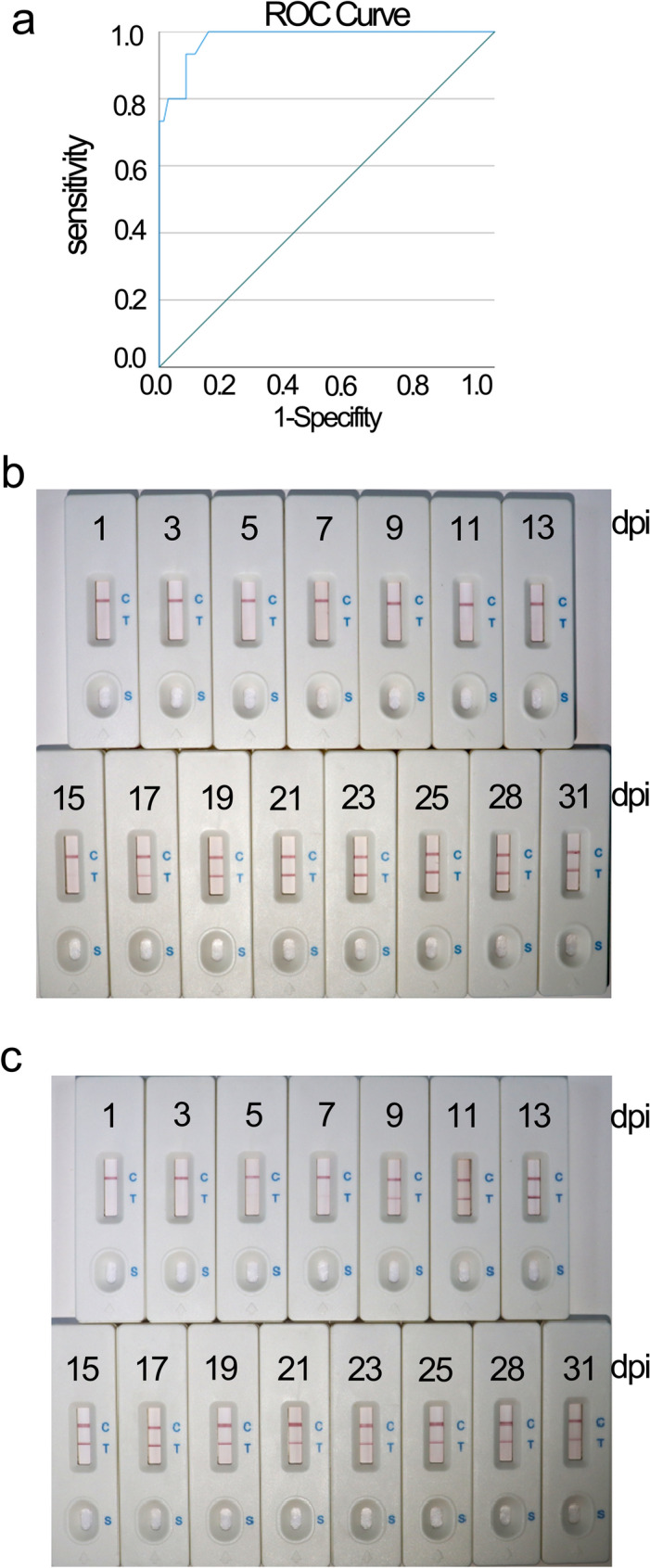
**a** ROC curve analysis between the AMA1C-GICA strips and commercial ELISA kit. The area under ROC curve (AUC) was 0.98. **b** Colloidal gold test strip assay for *Toxoplasma gondii* antibodies in the first rabbit serum from 1 to 31 dpi. **c** Colloidal gold test strip assay for *T. gondii* antibodies in the second rabbit serum from 1 to 31 dpi

The AMA1C-GICA strips were used to tested 90 cat serum samples, and the results were compared with those obtained using the commercial ELISA kit. In total, 15 positive sera were detected using the AMA1C-GICA strips and 15 positive sera were detected using the commercial ELISA kit (Table 1). Thus, the positive detection rate for the developed GICA strips and the commercial ELISA kit was the same (16.7%). When the commercial ELISA kit was used as the standard, the agreement rate was 93.3%. The ROC curves demonstrated that the relative sensitivity and the specificity were 85.3% and 92%, respectively, with an area of 98% [95% confidence interval (CI): 0.954–1.000, *P* < 0.0001] (Fig. 7a). The highest *T. gondii* antibody concentration used with cat serum was approximately 342.4 µg. A total of 66.7% (10/15) of the *T. gondii*-positive cats were female (Additional file 1: Table S3). A total of 60 rabbit serum samples were examined using the AMA1C-GICA strips and the commercial ELISA kit. As revealed in Figs. 3a, b and 7b, c, this technique accurately identified 20 positive sera. Weak immunogenic bands were displayed with sera at 15 dpi for the first rabbit and at 7 dpi for the second rabbit.

**Table 1:** Test results of 90 cat serum samples with AMA1C-GICA strips and commercial ELISA kit

		GICA^a^ strip		
		Positive	Negative	Total
ELISA^b^ Test	Positive	12	3	15
	Negative	3	72	75
	Total	15	75	90

^a^Gold immunochromatographic assay; ^b^ ELISA enzyme-linked immunosorbent assay

Then, a total of 347 serum samples from healthy cats and dogs were assessed to analyze anti-*T. gondii* antibody seropositivity rate in Jiaxing and Wenzhou, Zhejiang Province, China. Serum samples from 266 cats were tested, and 23.4% (11/47) and 7.8% (17/219) samples from in stray cats and domestic cats were positive for *T. gondii*, respectively. Serum samples from 81 domestic dogs were tested, and only one sample (1.2%) was positive for *T. gondii*; the serum antibody level was approximately 68.2 µg (Additional file 1: Table S4). Testing of 203 human specimens revealed that four were positive for antibodies to *T. gondii* (3.7% (4/108) in patients with schizophrenia and 0% (0/95) in healthy individuals) (Additional file 1: Table S5).

## Discussion

Toxoplasmosis has a long incubation period and subtle initial symptoms; moreover, it can lead to serious adverse effects in humans and infected pets [27]. Microscopy-based pathogen detection is the gold standard for the diagnosis of toxoplasmosis [28]; however, colloidal gold test strips have become an accepted and widely used point-of-care approach [29].

Several different *Toxoplasma* antigens, including toxoplasma surface antigen 2 (SAG2) [30], surface antigen 3 (SAG3) [31], dense granule antigen protein 7 (GRA7) [32–34], and rhoptry protein 14 (ROP14), are used for detecting *T. gondii* infection using GICA [26]. In our previous study, we identified AMA1 as a potential antigen for ELISA, but the high cutoff value and different form of this protein may lead to false positives when applied to GICA [20, 35]. By truncating AMA1, we found that AMA1C was more specific than full-length AMA1 and AMA1N. Therefore, AMA1C is a good target for the detection of *T. gondii.*

For sensitivity and specificity, each GICA strip has their own advantages. The sensitivity and specificity of the AMA1C-GICA strip were 85.3% and 92%, respectively. The N-terminal surface antigen 1 (SAG1) linked to granule antigen protein 2 (GRA2) was used as the test antigen, and the specificity of the test strips was 100% compared with the ELISA that used *T. gondii* whole-cell lysates as the antigen [36]. Using a circulating antigen, anti-ESA IgG was identified in 80 porcine sera, resulting in a sensitivity of 98% [37]. AMA1C-GICA strips offered advantages in terms of the detection limit. The AMA1C-GICA strips demonstrated a detection limit of 1:32 for *T. gondii*-positive serum, which was significantly better than the performance of either GRA7-GICA or TgROP14-GICA [26, 32–34].

IgM is typically serologically assayed 1 week after infection and is therefore considered an early and sensitive diagnostic marker for acute toxoplasmosis [16]. According to ELISA data, the earliest peaks of IgM in the two *T. gondii*-positive rabbits were observed at 7 and 15 dpi, whereas the earliest peaks of IgG were observed at 11 and 17 dpi. A weak TL was observed at 15 dpi in the first rabbit and at 7 dpi in the second rabbit, suggesting that AMA1C can be used for early detection of *T. gondii* infection. The interferon-gamma release assay (IGRA) can detect *T. gondii* infection as early as 4 dpi, while ELISA detects serum *T. gondii* IgM and IgG at 10 and 14 dpi, respectively. However, it is important to note that the ELISA-based IGRA protocol has notable limitations. Previous studies have recommended the use of purified antigens or synthesized peptides to improve its performance [38, 39]. In conclusion, GICA-based AMA1C represents a favorable option for early detection of *T. gondii* infection.

Notably, the AMA1C-GICA method is a dual-antigen sandwich assay that imposes fewer restrictions on the species origin of tested samples. Thus, this method was designed and developed into multispecies assay for toxoplasmosis for cats, dogs, humans, etc. Using the established method, we identified a positive rate of *T. gondii* antibodies of 23.4% (11/47) in stray cats, 7.8% (17/219) in domestic cats, and 1.2% in domestic dogs in Jiaxing and Wenzhou. Moreover, we also found a positive rate of *T. gondii* antibodies of 3.7% (4/108) in patients with schizophrenia and 0% (0/95) in healthy subjects in Lishui, China. However, the actual seroprevalence of *T. gondii* may vary considerably among different regions. For example, one study reported values of 2.5%–60% in cats, 0.6%–27.7% in dogs, and 0.7%–23.4% in humans in China [40]. Numerous recent studies have also indicated a significantly higher incidence of *T. gondii* infection in stray animals than in domestic animals [41–43]. In addition, the positive rate of 7.8% found in domestic cats implies that pet cats remain susceptible to *T. gondii* infection. This is significant, since pets can act as both carriers of the parasite and a direct cause of toxoplasmosis infection in humans [42]. Furthermore, a close association between *T. gondii* and schizophrenia is becoming increasingly clear, and *T. gondii* infection is therefore being recognized as a high-risk factor connected to psychiatric conditions [44, 45]. The measured prevalence of the anti-*T. gondii* antibody among patients with schizophrenia in eastern China was 17.98% [46], which was significantly higher than that observed in this study. Therefore, decreased seropositivity in patients with schizophrenia may be linked to hygiene conditions. The observation of anti-*T. gondii* antibodies may aid in the evaluation of the infection, facilitating the initiation of timely treatments. Thus, it is crucial to establish a dependable diagnostic method to minimize the spread of toxoplasmosis among definitive and other hosts in urban areas.

Although we constructed a standard curve for antibody concentrations, combining the color shades of the TL to determine the critical value is necessary. The T-value at this point may be similar to the ELISA cutoff value and may be more accurate for differentiating between negative and positive results. Furthermore, after quantifying or digitizing the test strips, negative, weak-positive, positive, and strong-positive antibody responses could be evaluated instead of just negative or positive responses. Such advancements make it easier to evaluate antibody levels and save time and money. To the best of our knowledge, this is the first time HMREADER has been used in conjunction with colloidal gold test strips to progress from qualitative to semiquantitative detection of antibody levels.

## Conclusions

We established a sensitive and highly specific GICA strip based on the truncated AMA1 protein (AMA1C) for the early detection of *T. gondii* in multiple species. HMREADER was used to qualitatively assess the test strip results, and the concentration of serum anti-*T. gondii* antibody could be intuitively obtained, providing data support for the next step of accurate treatment.

### Supplementary Information

**Additional file1: Table S1.** PCR primers for the construction of recombinant plasmids. **Table S2.** Different concentrations of AMA1 protein variants tested using ELISA with serum from four rabbits. **Table S3.** Test results of 15 serum-positive cat samples using AMA1C-GICA strips with HMREADER. **Table S4.** Test results of 14 serum-positive different animal samples using AMA1C-GICA strips with HMREADER. **Table S5.** Test results of four serum-positive human samples using AMA1C-GICA strips with HMREADER.

**Additional file 2: Figure S1.** Optimum pH and conjugate concentrations of mouse IgG. (a) The colloidal gold solution was adjusted to different pH values using 0.2 M K_2_CO_3_ to obtain an optimum pH value for mouse IgG. (b) A twofold series showing an increasing amount of mouse IgG added to a colloidal gold solution to identify the optimum conjugate amount of murine protein. **Figure S2.** Stability of the AMA1C-GICA strips. P: *Toxoplasma gondii*-positive control; N: *T. gondii*-negative control; PBS: blank control. **Figure S3.** Inter- and intra-batch variation of the AMA1C-GICA strips. P: *Toxoplasma gondii*-positive control, N: *T. gondii*-negative control.

## Data Availability

The gene sequences of AMA1 of *Toxoplasma gondii* generated and/or analyzed during this study are available in NCBI repository, GenBank: XM_002364813.1. The authors confirm that the data supporting the findings of this study are available within the article and/or its supplementary materials.

## Abbreviations

*T. gondii*: *Toxoplasma gondii*
*P. vivax*: *Plasmodium vivax*
*P. kellicotti*: *Paragonimus kellicotti*
*S. japonicum*: *Schistosoma japonicum*
*C. sinensis*: *Clonorchis sinensis*
*S. mansoni*: *Schistosoma mansoni*
GICA: Gold immunochromatographic assay
AMA1: Apical membrane antigen 1
AMA1C: C-terminal truncated apical membrane antigen 1
HMREADER: Portable colloidal gold immunochromatographic test strip analyzer
TL: Test line
CL: Control line
NC: Nitrocellulose
AMA1-iELISA: AMA1 indirect enzyme-linked immunosorbent assay
ELISA: Enzyme-linked immunosorbent assays
POCT: Point-of-care testing
HFFs: Human foreskin fibroblasts
DMEM: Dulbecco’s modified Eagle medium
FBS: Fetal bovine serum
PBS: Phosphate buffer saline
dpi: Days post infection
CAAS: Caprylic acid/ammonium sulfate precipitation
LB: Lysogeny broth
SDS‒PAGE: Sodium dodecyl sulfate-polyacrylamide gel electrophoresis
PBST: PBS with Tween-20
BSA: Bovine serum albumin
EP tubes: Eppendorf tubes
AUC: Area under the curve
ROC: Receiver operating characteristic
vLOD: Visual limit of detection
CI: Confidence interval
SAG1: Surface antigen 1
SAG2: Surface antigen 2
SAG3: Surface antigen 3
GRA2: Granule antigen protein 2
GRA7: Granule antigen protein 7
TgROP14: *T. gondii* Rhoptry protein 14
IGRA: Interferon-gamma release assay
ICT: Immunochromatography test
