# Supplementary material for: Application of gold immunochromatographic assay strip combined with digital evaluation for early detection of Toxoplasma gondii infection in multiple species
Source: Parasit Vectors. 2024 Feb 22;17:81. doi: 10.1186/s13071-024-06180-1 (PMC10882914; doi:10.1186/s13071-024-06180-1)
Supplement: Supplementary file 1 — Additional file1: Table S1. PCR primers for the construction of recombinant plasmids. Table S2. Different concentrations of AMA1 protein variants tested using ELISA with serum from four rabbits. Table S3. Test results of 15 serum-positive cat samples using AMA1C-GICA strips with HMREADER. Table S4. Test results of 14 serum-positive different animal samples using AMA1C-GICA strips with HMREADER. Table S5. Test results of four serum-positive human samples using AMA1C-GICA strips with HMREADER. [file 13071_2024_6180_MOESM1_ESM.docx]

**Table S1.** PCR primers for the construction of recombinant plasmids.

| Name | Sequence (5′–3′) ^a^ |
| --- | --- |
| AMA1C-F | GATATCCCGAATCAGGCACTGCGTG |
| AMA1C-R | ACGCGTCGACATAATCACCTTCAACC |
| AMA1N-F | GATATCACCAGCGGAAACCCATTTCAGG |
| AMA1N-R | ACGCGTCGACCGGACATTTGCTA |
| AMA1-F | GAATTCGATATCACCAGCGGAA |
| AMA1-R | CTCGAGGTCGACTTAATAATCACCTTCAA |

^a^Underlined letters of the primers represent the EcoRV and SalI restriction sites.

**Table S2.** Different concentrations of AMA1 protein variants were tested in ELISA with serum from four rabbits.

| Rabbit | AMA1C | AMA1N | AMA1 |  | AMA1C | AMA1N | AMA1 |
| --- | --- | --- | --- | --- | --- | --- | --- |
|  | 0.7µg/mL | | |  | 0.5µg/mL | | |
| 1 | 2.559 | 0.131 | 2.627 |  | 2.22 | 0.086 | 2.876 |
| 2 | 0.826 | 0.195 | 2.352 |  | 0.636 | 0.092 | 2.548 |
| 3 | 0.121 | 0.157 | 1.431 |  | 0.091 | 0.085 | 1.155 |
| 4 | 0.146 | 0.16 | 1.362 |  | 0.091 | 0.089 | 1.147 |

**Table S3.** Test results of 15 serum-positive cat samples by AMA1C-GICA strips with HMREADER.

| T-Values | Antibody content（µg） | Age（month） | Gender | Source |
| --- | --- | --- | --- | --- |
| 20 | 37.19 | 7 | Female | Domestic cat |
| 33 | 74.36 | 12 | Female | Domestic cat |
| 22 | 42.83 | 4 | Female | Domestic cat |
| 34 | 77.43 | 12 | Female | Domestic cat |
| 93 | 280.39 | 4 | Female | Domestic cat |
| 30 | 65.39 | 6 | Female | Stray cat |
| 93 | 280.39 | 5 | Unknown | Stray cat |
| 14 | 21.14 | 8 | Female | Domestic cat |
| 19 | 34.52 | 5 | Female | Domestic cat |
| 110 | 342.36 | Unknown | Female | Domestic cat |
| 16 | 26.49 | 6 | Male | Domestic cat |
| 16 | 26.49 | Unknown | Male | Domestic cat |
| 110 | 432.36 | 36 | Female | Domestic cat |
| 24 | 48.47 | 6 | Male | Domestic cat |
| 24 | 48.47 | 8 | Male | Domestic cat |

**Table S4.** Test results of 14 serum-positive different animal samples by AMA1C-GICA strips with HMREADER.

| T-Values | Antibody content（µg） | Age（month） | Gender | Source |
| --- | --- | --- | --- | --- |
| 31 | 37.19 | 4 | Male | Domestic dog |
| 28 | 59.75 | Unknown | Unknown | Stray cat |
| 44 | 108.15 | Unknown | Unknown | Stray cat |
| 45 | 111.22 | Unknown | Unknown | Stray cat |
| 29 | 62.57 | Unknown | Unknown | Stray cat |
| 14 | 21.14 | Unknown | Unknown | Stray cat |
| 16 | 26.49 | Unknown | Unknown | Stray cat |
| 25 | 51.29 | Unknown | Unknown | Stray cat |
| 20 | 37.19 | Unknown | Unknown | Stray cat |
| 23 | 45.65 | Unknown | Unknown | Stray cat |
| 22 | 42.83 | 48 | Female | Domestic cat |
| 48 | 120.44 | 12 | Female | Domestic cat |
| 29 | 65.57 | 48 | Male | Domestic cat |
| 29 | 65.57 | 72 | Male | Domestic cat |

**Table S5.** Test results of four serum-positive human samples by AMA1C-GICA strips with HMREADER.

| T-Values | Antibody content（µg） | Age（year） | Gender |
| --- | --- | --- | --- |
| 23 | 45.65 | 60 | Male |
| 21 | 40.01 | 48 | Male |
| 24 | 48.47 | 47 | Male |
| 45 | 111.22 | 29 | Female |
