# Supplementary figures and images for: Application of gold immunochromatographic assay strip combined with digital evaluation for early detection of Toxoplasma gondii infection in multiple species
Source: Parasit Vectors. 2024 Feb 22;17:81. doi: 10.1186/s13071-024-06180-1 (PMC10882914; doi:10.1186/s13071-024-06180-1)

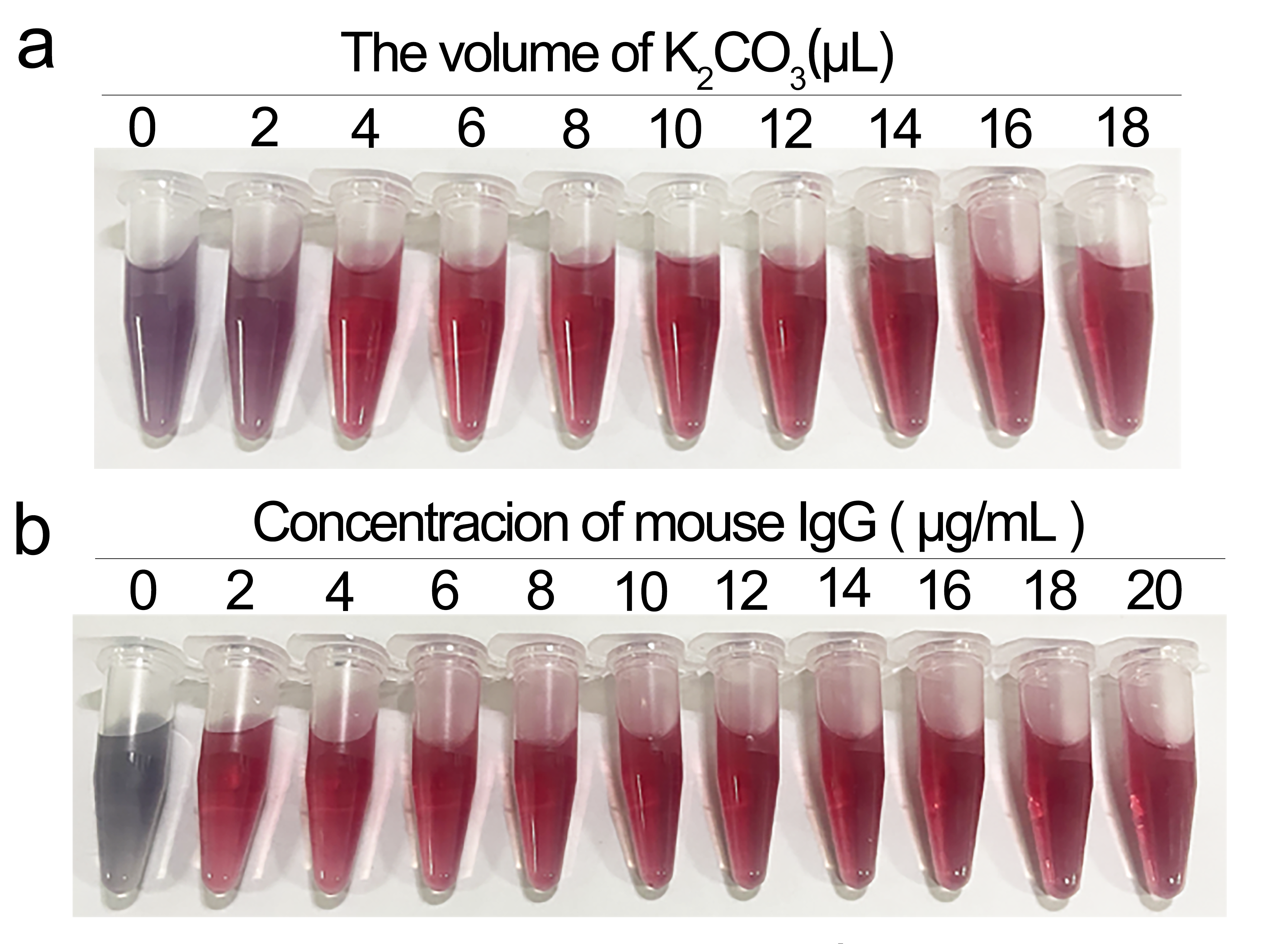

Supplement: Supplementary file 2 — Additional file 2: Figure S1. Optimum pH and conjugate concentrations of mouse IgG. (a) The colloidal gold solution was adjusted to different pH values using 0.2 M K2CO3 to obtain an optimum pH value for mouse IgG. (b) A twofold series showing an increasing amount of mouse IgG added to a colloidal gold solution to identify the optimum conjugate amount of murine protein. Figure S2. Stability of the AMA1C-GICA strips. P: Toxoplasma gondii-positive control; N: T. gondii-negative control; PBS: blank control. Figure S3. Inter- and intra-batch variation of the AMA1C-GICA strips. P: Toxoplasma gondii-positive control, N: T. gondii-negative control. [file 13071_2024_6180_MOESM2_ESM.zip › Additional file 2 Fig. S1.tif]

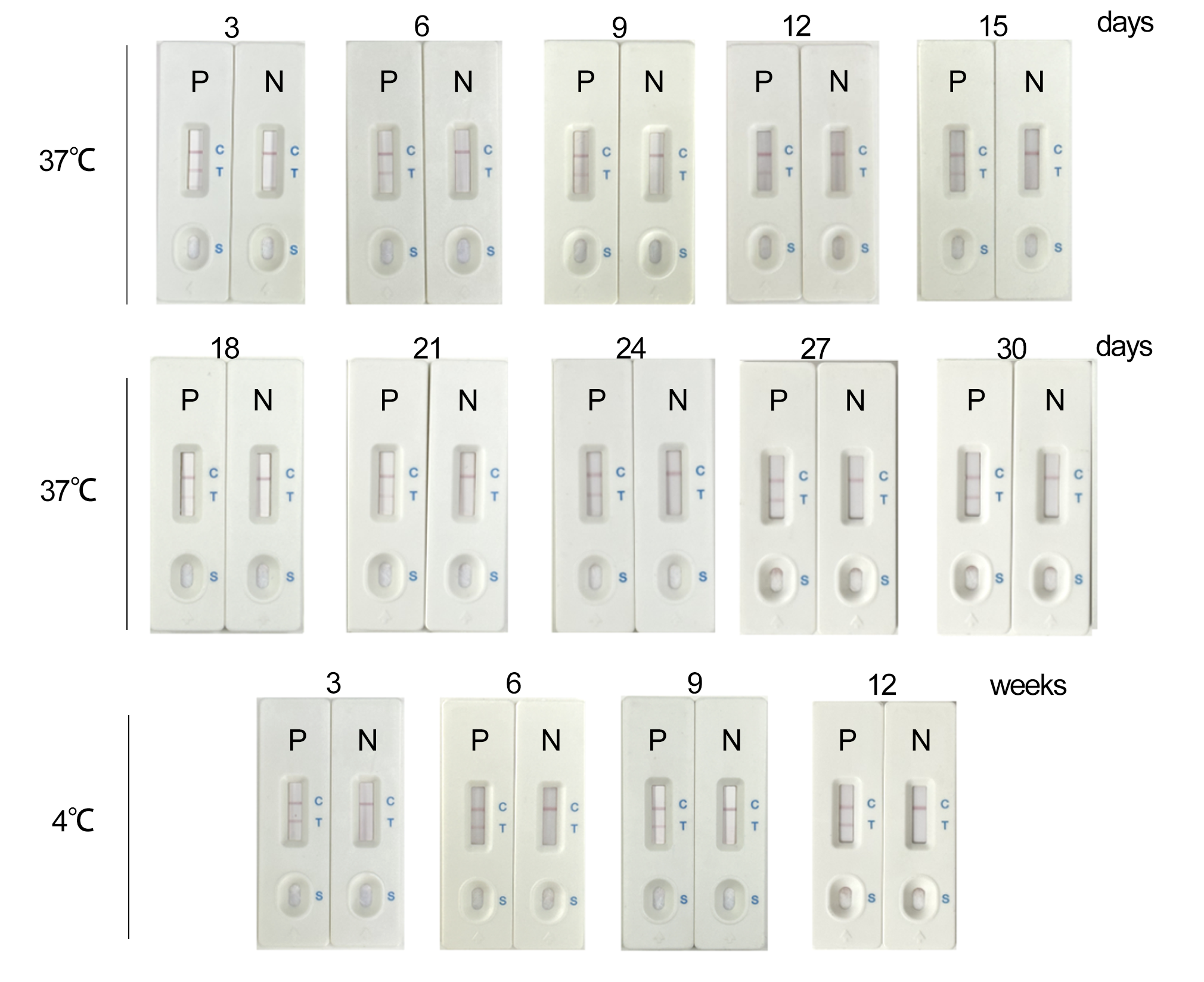

Supplement: Supplementary file 2 — Additional file 2: Figure S1. Optimum pH and conjugate concentrations of mouse IgG. (a) The colloidal gold solution was adjusted to different pH values using 0.2 M K2CO3 to obtain an optimum pH value for mouse IgG. (b) A twofold series showing an increasing amount of mouse IgG added to a colloidal gold solution to identify the optimum conjugate amount of murine protein. Figure S2. Stability of the AMA1C-GICA strips. P: Toxoplasma gondii-positive control; N: T. gondii-negative control; PBS: blank control. Figure S3. Inter- and intra-batch variation of the AMA1C-GICA strips. P: Toxoplasma gondii-positive control, N: T. gondii-negative control. [file 13071_2024_6180_MOESM2_ESM.zip › Additional file 2 Fig. S2.tif]

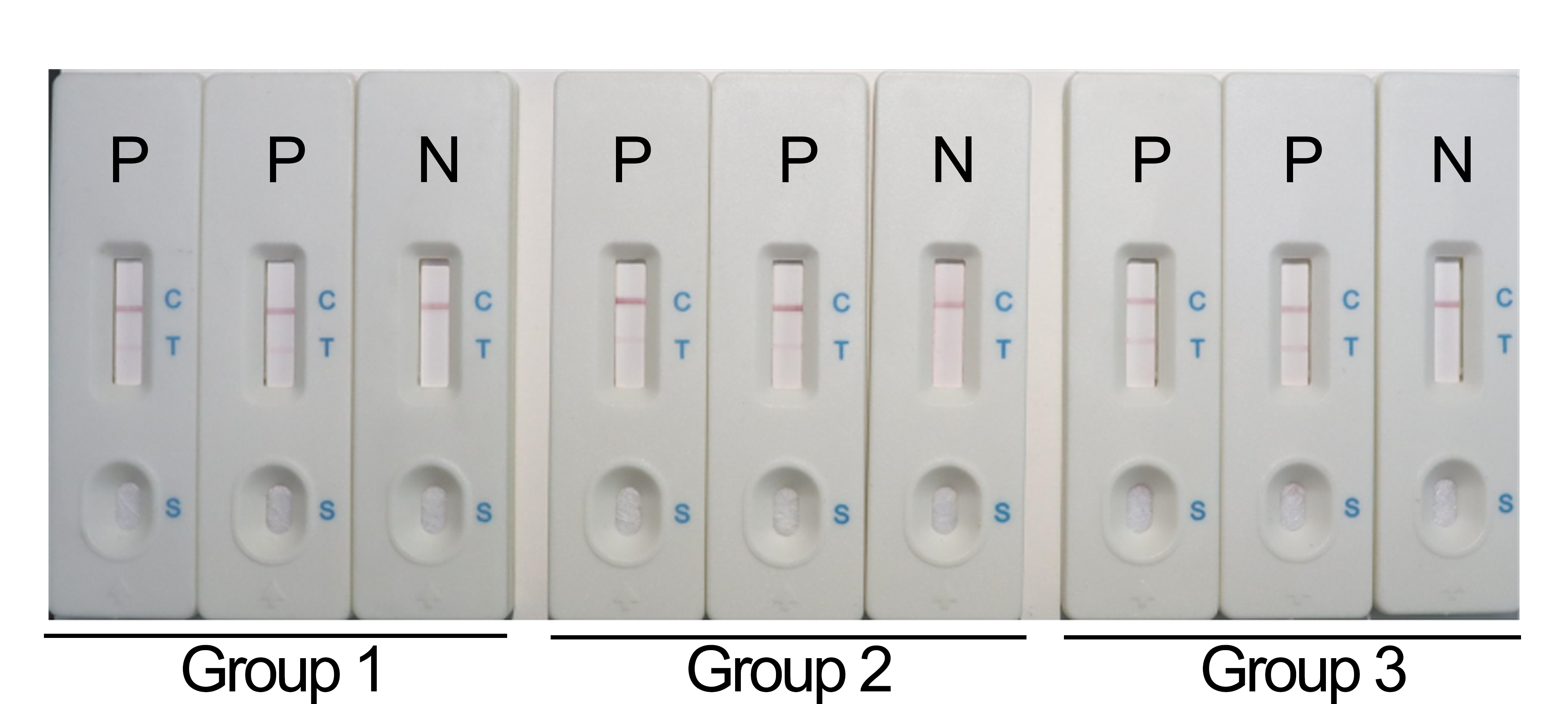

Supplement: Supplementary file 2 — Additional file 2: Figure S1. Optimum pH and conjugate concentrations of mouse IgG. (a) The colloidal gold solution was adjusted to different pH values using 0.2 M K2CO3 to obtain an optimum pH value for mouse IgG. (b) A twofold series showing an increasing amount of mouse IgG added to a colloidal gold solution to identify the optimum conjugate amount of murine protein. Figure S2. Stability of the AMA1C-GICA strips. P: Toxoplasma gondii-positive control; N: T. gondii-negative control; PBS: blank control. Figure S3. Inter- and intra-batch variation of the AMA1C-GICA strips. P: Toxoplasma gondii-positive control, N: T. gondii-negative control. [file 13071_2024_6180_MOESM2_ESM.zip › Additional file 2 Fig. S3.tif]
